# Assistive Technology among People with Dementia in Low Resource Settings

**DOI:** 10.1002/alz70858_105388

**Published:** 2025-12-25

**Authors:** Chiamaka Faith Okwudiri, Olufisayo Oluyinka Elugbadebo, Williams Nwagwu, Olusegun Baiyewu

**Affiliations:** ^1^ Department of Data and Information Science, University of Ibadan, Ibadan, Oyo State, Nigeria; ^2^ College of Medicine, University of Ibadan, Ibadan, Oyo, Nigeria; ^3^ Department of Data and Information Science, University of Ibadan, Ibadan, Oyo, Nigeria; ^4^ Department of Psychiatry, University of Ibadan, Ibadan, Oyo, Nigeria

## Abstract

Dementia is a leading cause of disability and dependence among older adults, particularly in low‐resource settings, where access to specialized care is often limited. The impact of dementia on individuals, families, and healthcare systems is profound, with significant social and economic implications. Assistive technologies (AT) offer a promising solution to support the independence, safety, and quality of life of people living with dementia (PLWD) and their caregivers. However, the adoption of AT remains relatively low, hindered by factors such as affordability, cultural perceptions, limited digital literacy, and infrastructural challenges.

This review describes the utilization of AT among people with dementia in sub‐Saharan Africa, focusing on the extent to which these technologies are used and the barriers to their adoption. It also evaluates the effectiveness of AT in improving patient outcomes and reducing caregiver burden in low‐resource settings. Additionally, it explores the potential for AT to improve dementia care in sub‐Saharan Africa, taking into account the unique socio‐economic and cultural dynamics of the region.

Despite being well‐established in high‐income countries and technologically advanced settings, the use of AT in dementia care remains underdeveloped in sub‐Saharan Africa. Some AT devices identified in low‐ and middle‐income countries (LMICs) include automated wheelchairs and GPS tracking systems. However, more advanced technologies, such as cognitive support devices and smart home technologies, remain scarce. This review emphasizes the urgent need for research and innovation in this field to adapt AT solutions to the cultural and socio‐economic contexts of low‐resource settings. By understanding and addressing the specific barriers to adoption, there is potential to improve the accessibility, affordability, and effectiveness of AT, ultimately enhancing the quality of dementia care and supporting caregivers in these regions.

